# Quantitative Depth Estimation in Lock-In Thermography: Modeling and Correction of Lateral Heat Conduction Effects

**DOI:** 10.3390/ma18225247

**Published:** 2025-11-20

**Authors:** Botao Ma, Shupeng Sun, Lin Zhang

**Affiliations:** Department of Engineering Mechanics, School of Civil Engineering, Shandong University, Jinan 250061, China; 202214991@mail.sdu.edu.cn (B.M.);

**Keywords:** lock-in thermography, lateral heat conduction, subsurface defect detection, blind frequency method, phase difference method

## Abstract

Lock-in thermography is a widely used nondestructive testing technique for detecting subsurface defects in solid materials. In this study, one-dimensional analytical modeling and three-dimensional finite element simulations were combined to elucidate how lateral heat conduction influences quantitative depth estimation in titanium alloy material using two inversion strategies: the blind frequency method and the phase difference method. Parametric analyses were conducted for defect radius-to-depth ratios ranging from 0.5 to 8 under various excitation frequencies. Results show that the blind frequency method can significantly underestimate defect depth with errors of up to 20.7% when the radius-to-depth ratio is as small as 0.5. To mitigate this bias, an exponential correction model was developed to compensate for lateral conduction effects, reducing the error to within ±5%. The accuracy of the phase difference method is found to depend jointly on defect depth, excitation frequency, and the ratio of defect radius to thermal diffusion length; estimation errors become negligible when this ratio exceeds 3. The novelty of this work lies in identifying lateral conduction as a key bias source and establishing a quantitative correction framework for the depth inversion based on the blind frequency method. The proposed approach is expected to enhance the accuracy of quantitative thermography for engineering applications.

## 1. Introduction

Nondestructive testing (NDT) plays a crucial role in materials science and engineering by enabling the detection of internal defects without compromising component integrity. It ensures the reliability, durability, and safety of critical structures in applications such as aerospace, automotive, and civil infrastructure [1,2,3]. Compared with other widely used nondestructive testing (NDT) techniques such as ultrasonic and eddy current testing, infrared thermography (IRT) offers distinct advantages for rapid, non-contact inspection over large surface areas. Ultrasonic testing provides excellent depth resolution but requires direct coupling with the specimen and extensive point-by-point scanning, which limits its efficiency for large geometries [4]. Eddy current testing is highly sensitive to surface and near-surface flaws in conductive materials, but is unsuitable for non-conductive materials and has limited penetration depth [5]. In contrast, thermography enables the detection of both surface and subsurface defects by monitoring temperature variations induced by thermal diffusion, allowing fast, full-field imaging without mechanical contact. Moreover, spatially resolved Lock-in thermography (LIT) can be used to quantitatively measure thermal diffusivity, further expanding its diagnostic capability [6,7,8,9]. These complementary features make thermography particularly attractive for inspecting metallic and composite structures in engineering applications. IRT is generally categorized into passive and active approaches. Passive thermography utilizes ambient or inherent thermal energy and is commonly employed in applications such as infrastructure monitoring and medical diagnostics [10,11]. In contrast, active thermography improves detection sensitivity by introducing external thermal excitation [12]. Due to its ability to reveal delamination, cracks, and corrosion, active thermography is extensively applied in evaluating composite materials, such as carbon fiber reinforced polymer (CFRP) and glass fiber reinforced polymer (GFRP) used in aerospace structures, including fuselages, wings, and engine components [13,14,15].

Active IRT techniques can be categorized into LIT, step heating, vibrothermography, and thermal-wave radar thermography [16,17,18,19,20,21,22,23,24], based on the type of thermal excitation. The theoretical principles underlying active thermography are well established in the foundational work by Almond and Patel [25], which provides a comprehensive treatment of photothermal wave phenomena and their application in nondestructive evaluation. Among these, LIT stands out for its robustness against non-uniform heating and surface emissivity variations, enabling effective suppression of background noise and enhancing the signal-to-noise ratio [26,27,28]. In LIT, a periodically modulated thermal excitation is applied to the surface of the specimen, and the resulting temperature response is analyzed in terms of phase and amplitude. The phase information, in particular, is sensitive to subsurface defects and allows for clear differentiation between defective and sound regions [29,30]. By tailoring the excitation frequency and analyzing the phase delay of thermal waves, LIT facilitates the extraction of depth-dependent information, leveraging the diffusive nature of thermal wave propagation. Furthermore, it becomes possible to quantitatively estimate the depth and size of subsurface defects by integrating multi-frequency measurements into analytical or numerical models, such as the blind frequency method or the phase difference method. This capability transforms LIT from a qualitative imaging tool into a quantitative evaluation technique [31,32,33].

In LIT, the penetration depth of thermal waves is governed by the thermal diffusion length, defined as $\mu =\sqrt{\alpha /\pi f}$, where α is the thermal diffusivity and f is the excitation frequency. By adjusting f, LIT enables the selective detection of defects at different depths, making it particularly effective for inspecting multilayer and composite materials in aerospace and civil engineering applications [18,34,35]. While a single sinusoidal excitation yields phase information corresponding to only one frequency, accurate quantitative depth analysis generally requires multi-frequency measurements [36]. By capturing thermal responses at various diffusion lengths, multi-frequency or broadband excitations improve both depth estimation and spatial resolution [35,37,38]. However, this approach can be time-consuming, especially when applied to large or thick specimens [39,40]. Hence, periodic excitation signals with rich spectral content, such as square waves or chirps, are often employed to enhance efficiency [35,38,41]. For data processing, frequency-domain demodulation is typically performed using lock-in amplifiers or discrete Fourier transforms [42,43,44].

In LIT, the quantitative estimation of defect depth typically relies on frequency-domain feature parameters. Among these approaches, the blind frequency method estimates defect depth by establishing an analytical relationship with the characteristic frequency, whereas the phase difference method infers depth based on the phase shift of surface thermal signals. Both methods have inherent limitations. The blind frequency method typically requires multiple measurements at different excitation frequencies to identify the characteristic blind frequency, which can be challenging to determine precisely without fine frequency tuning, even though low-duty-cycle square-wave lock-in thermography has been proposed to reduce measurement time successfully [45]. This process increases testing duration and may introduce uncertainty when the thermal response is weak or influenced by lateral heat conduction. On the other hand, the phase difference method relies on calibration or empirical modeling between the measured phase contrast and defect depth. The accuracy of both methods depends strongly on precise phase measurement and can be affected by surface emissivity variations, environmental noise, or material inhomogeneity. In detail, previous studies have shown that when the radius-to-depth ratio is small or when defects are located deep beneath the surface, the accuracy of these methods decreases significantly, primarily due to the effects of lateral heat conduction [46,47]. Efforts have been made to improve the performance of blind frequency and phase difference methods, such as incorporating numerical simulations or empirical corrections. However, these solutions are often tailored to specific materials and defect geometries, thereby limiting their generalizability [31,48]. Investigations on heterogeneous media, such as CFRP, have further confirmed that the interplay between lateral heat diffusion and anisotropic material properties has a significant impact on the reliability of depth quantification [32]. In recent years, novel strategies such as simultaneous multi-frequency excitation [49] and the sensitive frequency method based on thermal wave radar [50] have enhanced depth resolution. Moreover, machine learning methods [51,52], particularly those that integrate data-driven insights with physical modeling, have been investigated to improve automated defect detection and uncertainty quantification in infrared thermographic analysis [53]. Nevertheless, the quantitative estimation of defect depth remains a critical and yet unresolved challenge in LIT nondestructive testing. Developing accurate and physics-informed depth estimation frameworks remains a key research focus in the field.

To this end, this study targets the influence of lateral heat conduction on the accuracy of depth inversion in LIT. To achieve this, a hybrid framework combining a one-dimensional (1D) three-layer analytical thermal model with three-dimensional (3D) finite element simulations is developed to provide both theoretical and numerical insights. Two widely adopted methods, the blind frequency method and the phase difference method, are systematically analyzed under varying defect geometries and excitation frequencies. Quantitative relationships among the radius-to-depth ratio, thermal diffusion length, and phase response are established to clarify the role of lateral conduction in distorting depth estimates. Furthermore, a correction strategy for the blind frequency method is proposed, demonstrating a significant reduction in prediction bias across a range of conditions. Overall, this work not only enhances the fundamental understanding of lateral heat conduction effects in LIT but also provides practical correction guidelines to improve the quantitative accuracy of subsurface defect characterization for engineering applications.

## 2. Theory

**Figure 1:**
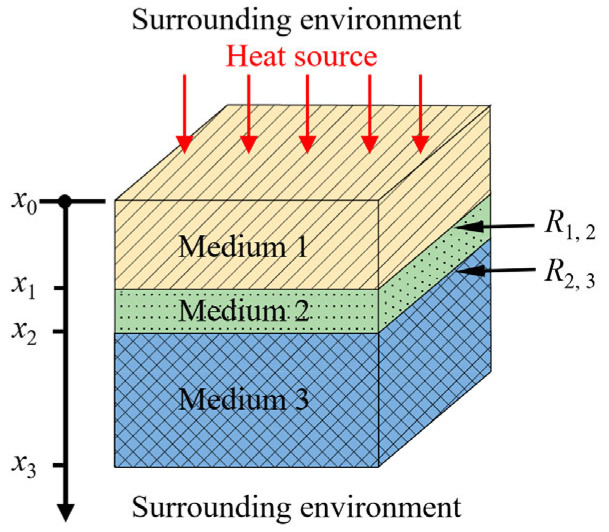
Schematic representation of the geometry of the three-layer model.

As illustrated in Figure 1, the 1D three-layer model is employed to approximate the thermal response of solid structures containing internal defects, which can provide valuable physical insight into the LIT. The structure consists of three layers of media, separated by planar interfaces, denoted by $x_{1}$ and $x_{2}$. The properties of each medium are defined as follows: thermal conductivity $k_{i}$, density $\rho_{i}$, specific heat $c_{i}$, and thermal diffusivity $\alpha_{i}=\frac{\kappa_{i}}{\rho_{i}c_{i}}$, where i = 1, 2, 3 corresponds to the respective layer. A sinusoidal heat source is applied on the top surface of Medium 1 and is expressed by $\frac{Q_{0}}{2}[1+\cos(\omega t)]$, and ω is the angular frequency. In this model, $h_{f}$ and $h_{r}$ denote the convective heat transfer coefficients on the front and rear external surfaces, respectively. $R_{1,2}$ and $R_{2,3}$ represent the interfacial thermal resistances between Medium 1 and 2, between Medium 2 and 3, respectively. It should be noted that this one-dimensional model considers heat transfer along the depth direction (*x*-axis).

Based on the boundary conditions and continuity requirements at the interfaces, the governing equations of the three-layer thermal model can be expressed in the following matrix form [54]:

(1) $$\left[\begin{array}{cccccc} k_{1}\sigma_{1}+h_{f} & -k_{1}\sigma_{1}+h_{f} & 0 & 0 & 0 & 0\\ -k_{1}\sigma_{1}\cdot \exp(-\sigma_{1}\cdot x_{1}) & k_{1}\sigma_{1}\cdot \exp(\sigma_{1}\cdot x_{1}) & k_{2}\sigma_{2}\cdot \exp(-\sigma_{2}\cdot x_{1}) & -k_{2}\sigma_{2}\cdot \exp(\sigma_{2}\cdot x_{1}) & 0 & 0\\ -\exp(-\sigma_{1}\cdot x_{1}) & -\exp(\sigma_{1}\cdot x_{1}) & \left(1+k_{2}\sigma_{2}R_{1,2})\cdot \exp(-\sigma_{2}\cdot x_{1}\right) & \left(1-k_{2}\sigma_{2}R_{1,2})\cdot \exp(\sigma_{2}\cdot x_{1}\right) & 0 & 0\\ 0 & 0 & -\exp(-\sigma_{2}\cdot x_{2}) & -\exp(\sigma_{2}\cdot x_{2}) & \left(1+k_{3}\sigma_{3}R_{2,3})\cdot \exp(-\sigma_{3}\cdot x_{2}\right) & \left(1-k_{3}\sigma_{3}R_{2,3})\cdot \exp(\sigma_{3}\cdot x_{2}\right)\\ 0 & 0 & -k_{2}\sigma_{2}\cdot \exp(-\sigma_{2}\cdot x_{2}) & k_{2}\sigma_{2}\cdot \exp(\sigma_{2}\cdot x_{2}) & k_{3}\sigma_{3}\cdot \exp(-\sigma_{3}\cdot x_{2}) & -k_{3}\sigma_{3}\cdot \exp(\sigma_{3}\cdot x_{2})\\ 0 & 0 & 0 & 0 & \left(k_{3}\sigma_{3}-h_{r})\cdot \exp(-\sigma_{3}\cdot x_{3}\right) & \left(-k_{3}\sigma_{3}-h_{r})\cdot \exp(\sigma_{3}\cdot x_{3}\right) \end{array}\right]\left[\begin{array}{c} A_{1}\\ B_{1}\\ A_{2}\\ B_{2}\\ A_{3}\\ B_{3} \end{array}\right]=\left[\begin{array}{c} \frac{Q_{0}}{2}\\ 0\\ 0\\ 0\\ 0\\ 0 \end{array}\right]$$

where $\sigma_{i}=(1+j){\left(\frac{\omega }{2\alpha_{i}}\right)}^{\frac{1}{2}}$. Based on the matrix formulation presented above, the temperature response at the top surface can be obtained. Specifically, the alternating current amplitude and phase of the thermal signal are given by

(2) $$T_{a1}=A_{1}+B_{1}$$

(3) $$\Phi =\arg(A_{1}+B_{1})$$

Here, $A_{1}+B_{1}$ is also a complex quantity. Φ is the phase angle of, i.e., the phase difference between the surface temperature and heat source. This analytical solution provides a baseline for predicting surface temperature phase response and will serve as the theoretical foundation for the blind frequency method and phase difference method discussed in later sections.

## 3. Methods

### 3.1. Theoretical Analysis of Defect Depth Prediction Methods

The analytical formulation expressed in Equations (1)–(3) was employed to determine the surface temperature response. Building on this formulation, a theoretical analysis was conducted for two widely used defect depth estimation techniques: the blind frequency method and the phase difference method. Within the framework of the 1D three-layer model, the temperature response of the system under LIT can be precisely computed for given thermal and geometrical parameters, assuming negligible lateral heat conduction. The temperatures of the defective and defect-free regions can be evaluated separately by assigning the material properties of air to the defects. Let $\varphi_{d}$ denote the phase of the defective region and $\varphi_{\mathrm{free}}$ represent that of the defect-free region, the phase difference is then expressed as

(4) $$\Delta \varphi =\varphi_{\mathrm{free}}-\varphi_{d}$$

**Figure 2:**
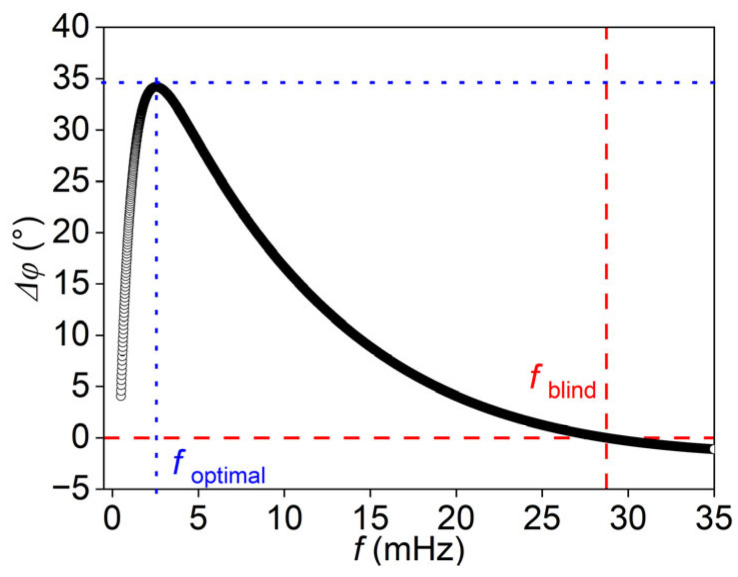
The analytical solutions for the defect at a depth of 8 mm [54].

In detail, the results were calculated for a titanium alloy model with a total thickness of 30 mm. In the defective model, the material parameters for the second plate were set to air, and all parameters are listed in Table 1. The defective region had a depth of 8 mm and a thickness of 4 mm, and the interfacial thermal resistances were zero. Sinusoidal thermal waves with frequencies ranging from 0.5 to 35 mHz were applied as heat sources, using a step size of 0.01 mHz. The phase differences were calculated based on Equations (1)–(4), and the results shown in Figure 2 illustrate how the phase difference varies with the excitation frequency. The blind frequency, defined as the zero-crossing point of the phase difference–frequency curve, was found to be 28.74 mHz.

**Figure 3:**
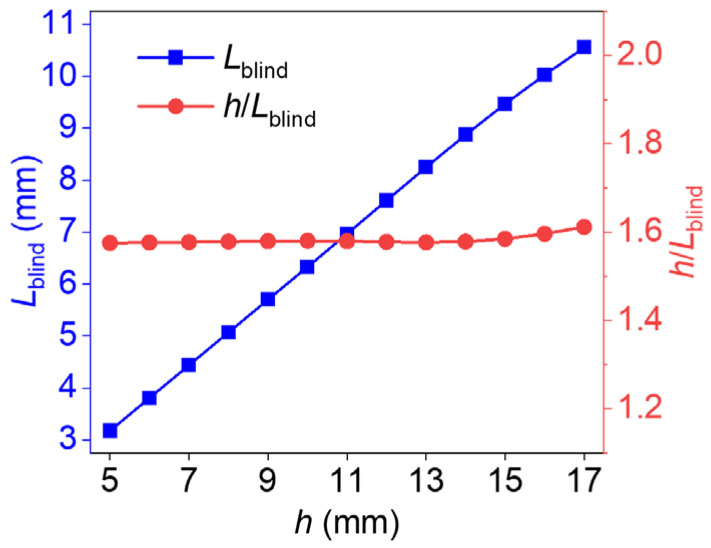
Thermal diffusion lengths at blind frequencies for various defect depths.

The blind frequency method establishes the relationship between defect depth and the corresponding blind frequency. First, the phase difference–frequency curves shown in Figure 2 were obtained from theoretical models with defect depths ranging from 5 mm to 17 mm. The blind frequency for each case was then identified from the zero-crossing point of the phase difference curve. Subsequently, the thermal diffusion length at the blind frequency ($L_{\mathrm{blind}}$) was calculated by $L_{\mathrm{blind}}=\sqrt{\alpha /\left(\pi f_{\mathrm{blind}}\right)}$, where α is the thermal diffusivity and $f_{\mathrm{blind}}$ is the identified blind frequency. This allowed the relationship between $L_{\mathrm{blind}}$ and defect depth to be established, as illustrated by the blue line in Figure 3. Notably, the ratio of $L_{\mathrm{blind}}$ to the defect depth remains nearly constant across all cases, as highlighted by the red line in the figure. In this way, as the blind frequencies of the defects are determined by LIT, the defect depth can be predicted with the ratio of the thermal diffusion length at the blind frequency to the defect depth. This method is named the blind frequency method in this study, and the ratio is found to be approximately 1.59.

**Figure 4:**
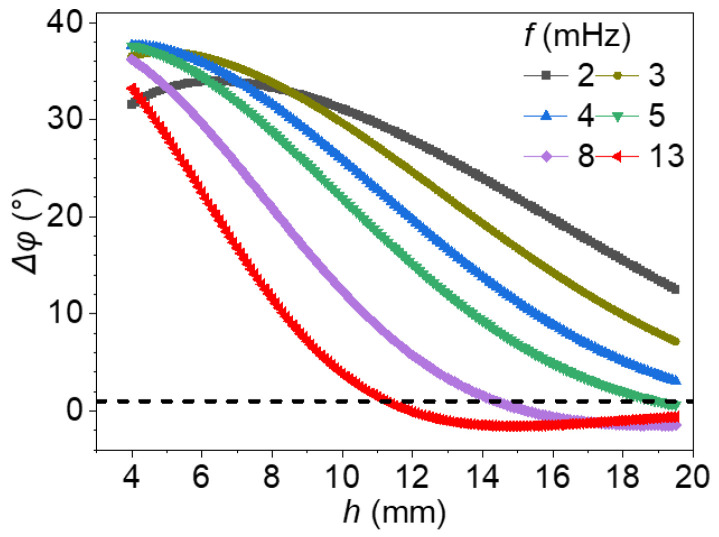
Phase difference versus defect depth under different excitation frequencies.

Figure 4 illustrates the theoretical relationships between phase difference (Δφ) and defect depth under various excitation frequencies ranging from 2 mHz to 13 mHz. For each excitation frequency, a distinct Δφ−h curve is obtained by varying the defect depth in the model. Across all frequencies, Δφ generally decreases with increasing depth, reflecting the attenuation of modulated thermal waves as they propagate into the material. However, the shape and steepness of each curve vary significantly with frequency, revealing important frequency-dependent behaviors relevant to depth estimation.

At low frequencies, such as 2 mHz and 3 mHz, the phase difference exhibits a broad and gradual decline over a wide depth range. In particular, for 2 mHz, Δφ remains above 12° even at a depth of 19.5 mm, indicating intense thermal wave penetration and reliable detection of deep-seated defects. However, the relatively flat slope near the surface implies limited sensitivity for distinguishing shallow defects. In contrast, at higher frequencies such as 13 mHz, the phase response is much sharper in the near-surface region. The Δφ curve drops rapidly within the first few millimeters and approaches zero beyond approximately 11–12 mm (as indicated by the horizontal dashed line at Δφ = 1°), making it highly sensitive for surface or near-surface defects but unsuitable for deeper targets due to the loss of phase contrast and increased ambiguity. In this case, a single measured phase difference value may correspond to a wide range of depths, reducing inversion accuracy. Intermediate excitation frequencies, particularly 4 mHz and 5 mHz, offer a practical compromise. These curves are monotonic and moderately sloped, spanning both shallow and moderate depth ranges. This behavior ensures sufficient phase contrast across depths while maintaining quantitative reliability. Additionally, a gently varying, near-linear Δφ–depth relationship at these frequencies helps minimize error propagation. Slight variations or noise in measured phase difference values lead to minor deviations in estimated depth.

Taken together, these trends highlight a critical trade-off in frequency selection: lower frequencies enable deeper penetration but with reduced gradient sensitivity; higher frequencies enhance near-surface resolution but suffer from limited depth reach. Therefore, the choice of excitation frequency should be strategically tailored to the expected range of defect depths. Intermediate frequencies provide an optimal balance, supporting robust and accurate depth quantification across a broad range of defect depths. Furthermore, an excitation frequency yielding a steep Δφ–depth gradient is desirable, as it reduces the sensitivity of depth estimation to measurement noise and improves overall accuracy.

### 3.2. Finite Element Simulations for Thermal Response

**Figure 5:**
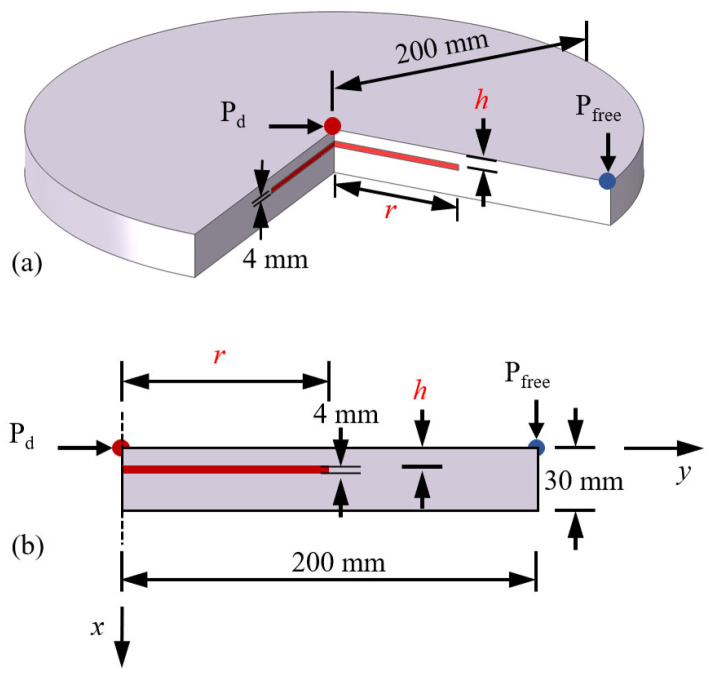
(**a**) Illustrative cutaway view of the finite element model with the defect highlighted in red, (**b**) the axisymmetric section view of the model with the defect shown in red.

Although the 1D model provides valuable analytical insights, it inherently neglects the effects of lateral heat conduction. To overcome this limitation, 3D finite element models were developed to investigate how lateral thermal conduction influences the correlation between defect depth and thermal diffusion length. As illustrated in Figure 5a, a three-quarter cutaway view of the finite element model reveals the internal defect within the cylindrical plate. Both the plate and the embedded defect are modeled with axisymmetric cylindrical geometry. Figure 5b presents an axisymmetric section view of the model, where the dimensions are annotated: the plate radius is 200 mm, the total thickness is 30 mm, and the defect thickness is 4 mm. The two key geometric variables, *h* and defect radius (*r*), are represented in the schematic. By systematically varying these two parameters, a set of finite element models was constructed to explore how defect geometry affects the resulting thermal response. The specific combinations of defect depth, radius, and the corresponding radius-to-depth ratio (*r*/*h*) are summarized in Table 2 for the subsequent quantitative analysis.

Finite element simulations were conducted using ABAQUS 2020. Given the axisymmetric geometry of the model as shown in Figure 5b, axisymmetric elements were employed for high computational efficiency. The vertical dashed line indicates the axis of symmetry. The thermal response analysis was carried out using the DCAX4 elements, which are four-node axisymmetric heat transfer elements. A uniform mesh size of 0.5 mm was applied to all models. For thermal signal evaluation, the point at the upper right corner of the model, defined as $P_{\mathrm{free}}$ and located farthest from the defect, was used to represent the defect-free temperature response. In contrast, the point at the upper left corner, defined as $P_{d}$ and aligned with the center of the defect, was selected to assess the temperature variation induced by the defect. The defect-free regions were assigned the thermal properties of titanium alloy, while the defective regions were modeled as air to represent voids. All material properties [54] used in the simulations are summarized in Table 1.

The initial temperature of the model was set to 20 °C. Convective boundary conditions were applied to both the top and bottom surfaces, with the convective heat transfer coefficient set to 30 $W/\left(m^{2}\cdot K\right)$. A thermal wave, characterized by a 5% duty cycle, a period of 2000 s (corresponding to a frequency of 0.5 mHz), and a power of 10 kW, was applied to the top surface of the model. This square wave contains harmonics at multiple frequencies, integer multiples of the fundamental frequency, and the harmonic amplitude is zero only at frequencies with integer multiples of 20 times the fundamental frequency. A total of six wave cycles were applied to the model, with the increment size fixed at 0.2 s throughout the simulation. This study was conducted within a deterministic finite element framework, in which all input parameters were predefined and systematically controlled. As a result, statistical uncertainty analysis was not included in the current scope. Convergence analyses were performed with respect to both mesh density and time-step size, confirming that the phase and temperature results were independent of the discretization parameters.

**Figure 6:**
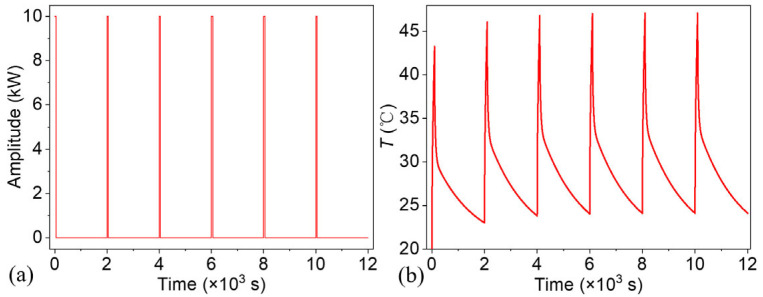
(**a**) Thermal wave and (**b**) temperature response of the defect-free point $P_{\mathrm{free}}$ 54.

Figure 6 shows the thermal wave and temperature response of the point P_free_ at the upper right corner of the top surface. The temperature field obtained from time-domain finite element simulations under square-wave excitation inherently includes multiple harmonic components due to the spectral characteristics of the input signal. Consequently, the simulated thermal response reflects the combined contributions of the fundamental frequency and its higher-order harmonics. Owing to their higher frequencies, these harmonic components possess shorter thermal diffusion lengths, making them particularly sensitive to shallow, near-surface regions. They locally modulate the temperature distribution and enhance phase gradients associated with surface-level defects. In contrast, the fundamental frequency, with its longer thermal diffusion length, governs the thermal behavior at greater depths, contributing to the deeper defect response. This broadband excitation approach effectively captures a broader spectrum of thermal behavior, eliminating the need for multiple simulations at discrete sinusoidal frequencies. To extract frequency-specific phase information, including both the fundamental and higher harmonics, the simulated time-domain temperature signal can be post-processed using Fourier transform techniques, enabling further frequency-domain analysis of the thermal response.

**Figure 7:**
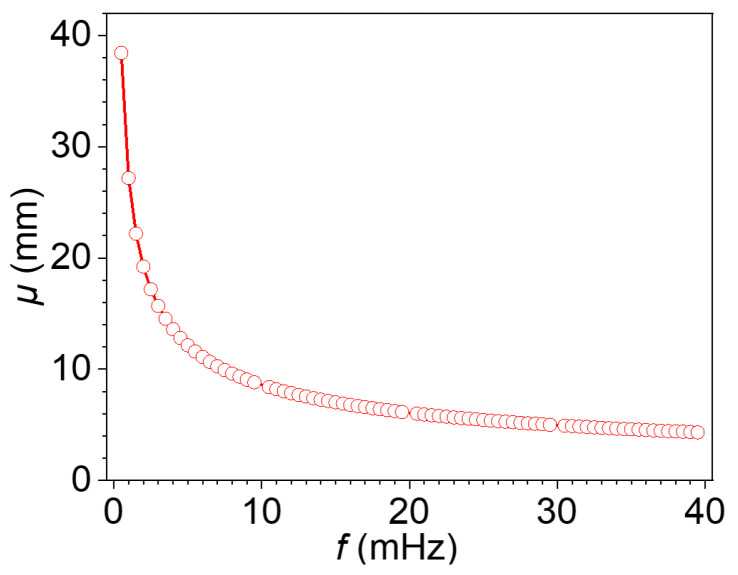
The thermal diffusion lengths corresponding to the harmonics of the square wave.

Figure 7 presents the thermal diffusion lengths associated with the harmonic components of the square-wave excitation, highlighting the depth-dependent penetration behavior of thermal waves across the frequency spectrum. Since a square wave contains a wide range of odd harmonics, it enables simultaneous probing of subsurface defects at multiple depths. This figure provides an intuitive understanding of the multi-frequency thermal diffusion behavior. It serves as a physical foundation for the subsequent application of the blind frequency method and the phase difference method. It also helps elucidate the influence of lateral heat conduction on the accuracy of depth estimation.

In this study, modulated thermal waves were applied to the models listed in Table 2, and the temporal temperature responses at nodes on the top surface of each model were recorded. The temperature responses from the final excitation cycle were recorded after the system reached steady-state. These time-domain signals were then processed using the Discrete Fourier Transform (DFT) to extract the amplitude and phase components at each harmonic frequency. In the following section, these frequency-domain characteristics, with particular emphasis on the phase differences between defective and defect-free points, will be employed to assess the accuracy of two depth inversion methods: the blind frequency method and the phase difference method. By systematically comparing simulation results against analytical predictions, the influence of lateral heat conduction on the reliability of subsurface defect depth estimation is quantified.

## 4. Results and Discussion

This section investigates the influence of lateral heat conduction on the accuracy of defect depth inversion in LIT. Two commonly used methods, the blind frequency method and the phase difference method, are analyzed through combined analytical and numerical approaches. The effects of defect radius, depth, excitation frequency, and geometric ratios are systematically examined. A correction model is proposed to enhance the accuracy of the blind frequency method, while the limitations of applying a universal correction to the phase difference method are also discussed. First, the blind frequency method is evaluated under varying radius-to-depth ratios, and an exponential bias-correction model is developed to compensate for deviations caused by lateral heat conduction. Then, the phase difference method is examined to investigate how defect geometry and excitation frequency jointly influence depth estimation accuracy, revealing a strong dependence on frequency and highlighting the complex role of lateral heat conduction. These findings provide a quantitative foundation for improving defect depth estimation accuracy and underscore the limitations of conventional 1D analytical models when applied to real-world thermographic inspections.

### 4.1. Analysis of the Effect of Radius on the Blind Frequency Method

**Figure 8:**
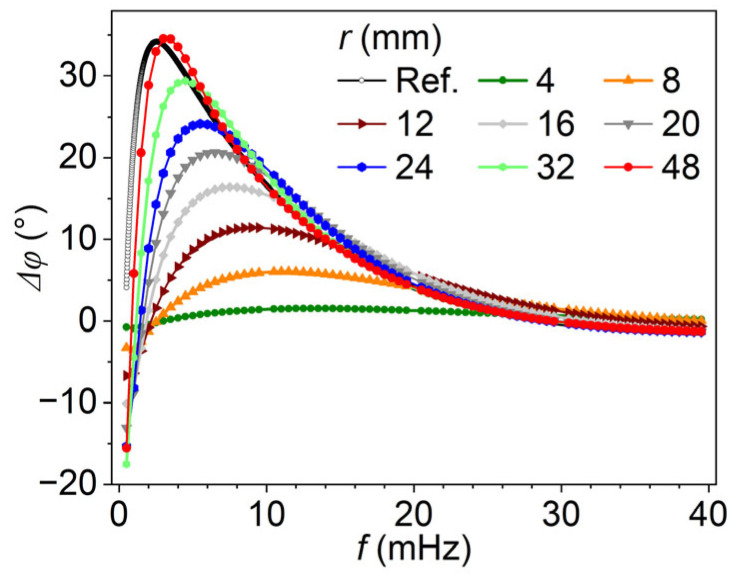
Phase difference–frequency curves of the model for different defect radii.

Figure 8 presents the relationship between phase difference (*Δφ*, in degrees) and excitation frequency (*f*, in mHz) for models with varying defect radii (*r*, in mm). The defect depth is held constant at 8 mm, while the radius ranges from 4 mm to 48 mm, as indicated by the legend. The black curve labeled Ref. represents the analytical 1D reference solution, serving as a baseline for comparison. At lower frequencies (below 10 mHz), all models except for the one with a radius of 4 mm exhibit a characteristic peak in phase difference, followed by a gradual decay as frequency increases. Notably, the peak value and the overall shape of the *Δφ-f* curves are strongly influenced by the defect radius. For smaller radii (e.g., from 8 to 12 mm), the phase difference remains significantly lower than the reference curve, indicating substantial influence from lateral heat conduction. As the defect radius increases, the phase curves shift upward and leftward and become increasingly aligned with the 1D analytical solution, demonstrating a transition toward 1D-like thermal behavior. For large radii (e.g., from 32 to 48 mm), the phase response closely matches the reference curve across the frequency range, suggesting that lateral conduction effects become negligible at these sizes of the defect center. These trends confirm that defect geometry, particularly the radius-to-depth ratio, plays a critical role in the frequency-dependent phase response and ultimately affects the accuracy of depth inversion methods such as the blind frequency method.

**Figure 9:**
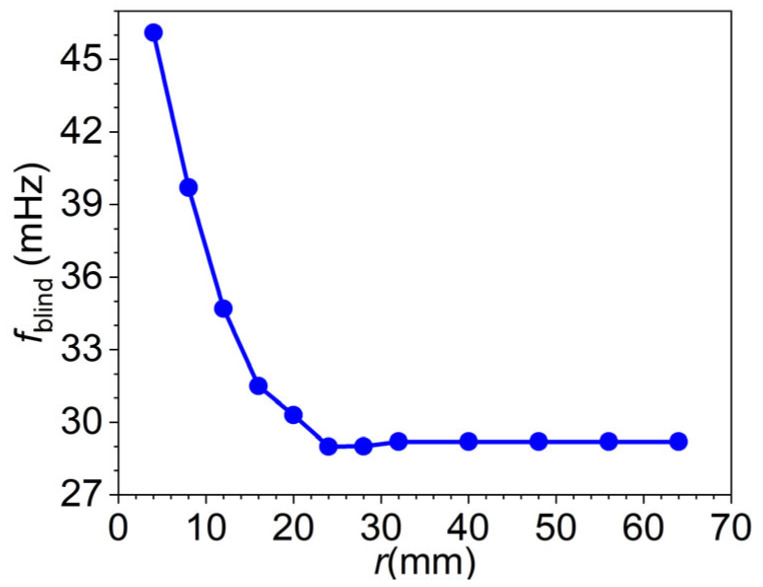
Blind frequencies for models of different radii at a fixed depth of 8 mm.

The blind frequency corresponding to each model was extracted from the phase difference–frequency curves. As shown in Figure 9, the blind frequency decreases with increasing defect radius and gradually converges toward the analytical value of 28.74 mHz. This behavior indicates that for small-radius defects, lateral heat conduction significantly elevates the blind frequency relative to the 1D theoretical prediction. As the defect radius increases, the influence of lateral conduction diminishes, and heat transfer at the defect center becomes increasingly one-dimensional in nature. As a result, the blind frequency progressively aligns with the theoretical value predicted by the 1D analytical model, confirming that geometric effects, particularly radius-to-depth ratio, play a critical role in the accuracy of depth estimation based on blind frequency.

**Figure 10:**
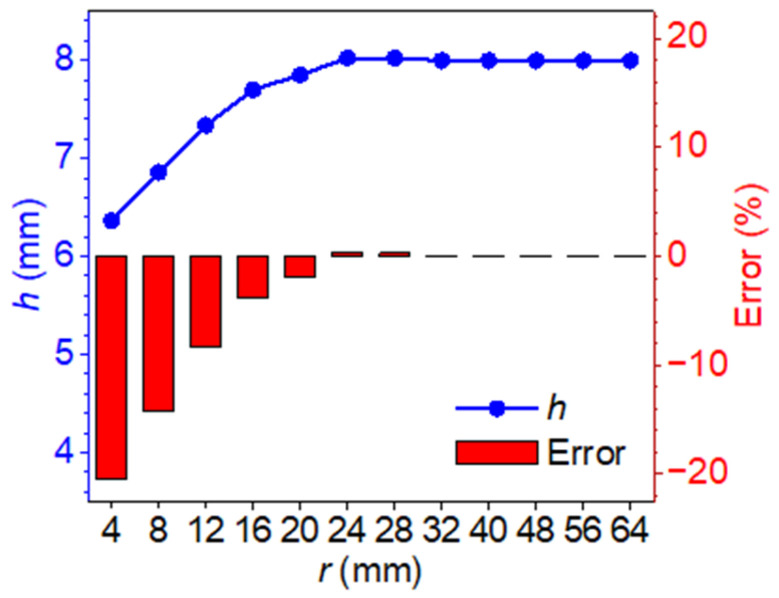
Defect depth obtained through the blind frequency method and error.

Once the blind frequency is identified, the corresponding thermal diffusion length can be calculated. According to the analytical model, the defect depth is approximately 1.59 times the thermal diffusion length associated with the blind frequency. Using this relationship, defect depths were predicted for various models, and the associated estimation errors were quantified. As shown in Figure 10, the prediction error is most significant when the defect radius is 4 mm, reaching −20.47%. As the defect radius increases, the error systematically decreases and approaches zero when the radius reaches 24 mm. These findings demonstrate that defect radius is a critical parameter affecting the depth estimation accuracy of the blind frequency method. For small-radius defects, lateral heat conduction has a significant impact on the thermal response, resulting in a noticeable shift in the blind frequency and, consequently, substantial prediction errors. In contrast, when the defect radius is sufficiently large, lateral conduction effects become negligible, and the blind frequency aligns closely with the theoretical prediction based on the 1D model.

To further investigate the influence of defect geometry, additional simulations were conducted by systematically varying defect depth and radius, while keeping all other model parameters identical to those described in Section 3.2. In these cases, *r*/*h* was held constant, and the specific combinations of defect depth and radius are listed in Table 2.

The resulting phase difference–frequency curves exhibit trends consistent with the case at 8 mm depth. As the defect radius increases, the curves converge progressively toward the 1D analytical solution, indicating a reduction in lateral heat conduction effects. Additionally, increasing the defect depth results in an apparent decrease in the maximum phase difference, reflecting greater attenuation of thermal waves with depth. The corresponding blind frequencies, extracted from these curves and summarized in Table 3, follow a consistent trend. The blind frequency decreases with increasing radius. It gradually converges to the analytical value predicted by the 1D model.

**Figure 11:**
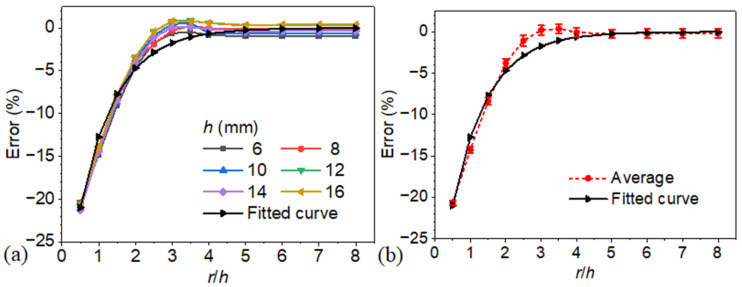
(**a**) Depth prediction error based on blind frequency for varying radius-to-depth ratios. (**b**) Relationship between average depth estimation error and the radius-to-depth ratio.

A detailed analysis was conducted on models with *r*/*h*, and the results are presented in Figure 11. As shown in panel (a), the defect depth prediction error, derived from the blind frequency method, is strongly dependent on the ratio of *r*/*h*. Figure 11b illustrates the relationship between average depth estimation error and the radius-to-depth ratio (*r*/*h*). The error trend follows an exponential pattern, indicating that prediction accuracy decreases significantly as *r*/*h* becomes smaller. This behavior is consistent with the results in Figure 11a, where similar trends are observed across different defect depths, further confirming the adverse impact of lateral heat conduction at low *r*/*h* values. As the ratio increases, the error gradually decreases and becomes negligible once the ratio exceeds 3.0. This stabilization of prediction error across models with different absolute defect depths suggests a geometrically driven scaling behavior. As illustrated in Figure 3, the thermal diffusion length at the blind frequency is approximately proportional to the defect depth. Since this diffusion length governs lateral heat conduction, its effect on the blind frequency and consequently on the depth estimation error also scales proportionally with depth. Therefore, when the radius-to-depth ratio is held constant, the impact of lateral conduction remains consistent, resulting in nearly identical prediction errors across defects of varying depths.

The variation in detection error exhibits strong consistency when expressed as a function of the radius-to-depth ratio (ε=r/h), as shown in Figure 11. Leveraging this convergence behavior, an empirical correction model is proposed for the relative bias E(ε) in the blind frequency-based depth estimate, formulated through an exponential fit:

(5) $$E(\varepsilon )=-0.21e^{-(\varepsilon -0.5)}$$

The exponential decay form represents the gradual reduction in lateral heat conduction effects as the defect radius-to-depth ratio increases. When this ratio is small, a substantial portion of the heat flux is diverted laterally around the defect instead of propagating purely in the through-thickness direction. This enhanced lateral spreading reduces the phase contrast between defective and defect-free regions, causing the surface phase response to deviate from the one-dimensional prediction and leading to underestimation of defect depth. As the defect radius increases, the heat flow at the defect center becomes increasingly one-dimensional, and the bias introduced by lateral conduction diminishes. The correction model captures this attenuation behavior and restores the intrinsic relationship between blind frequency and defect depth, improving the physical accuracy and interpretability of depth inversion results.

**Figure 12:**
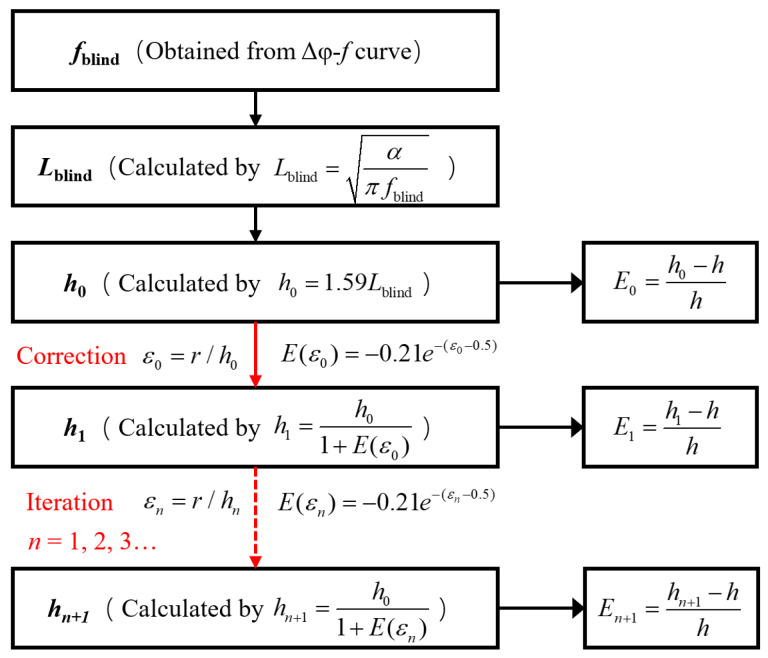
Correction workflow for blind frequency–based depth estimation.

Figure 12 illustrates the proposed empirical correction workflow for defect depth estimation using the blind frequency method. Here, the defect radius (*r*) must be known in advance. Starting from the extracted blind frequency (*f*_blind_), the thermal diffusion length is computed and used to obtain an initial depth estimate (*h*_0_), and the error is denoted as *E*_0_. Based on the radius and the predicted *h*_0_, the ratio $\varepsilon_{0}=r/h_{0}$ is evaluated, and a relative bias is evaluated with Equation (4), yielding a refined depth estimate (*h*_1_)

(6) $$h_{1}=\frac{h_{0}}{1+E(\varepsilon_{0})}$$

A second correction iteration can be performed to reduce residual error further, resulting in a refined value (*h*_2_). Error metrics at each step (*E*_0_, *E*_1_, *E*_2_) quantify the deviation from the true depth, demonstrating rapid convergence and high accuracy of the proposed approach.

(7) $$h_{2}=\frac{h_{0}}{1+E(\varepsilon_{1})}$$

**Figure 13:**
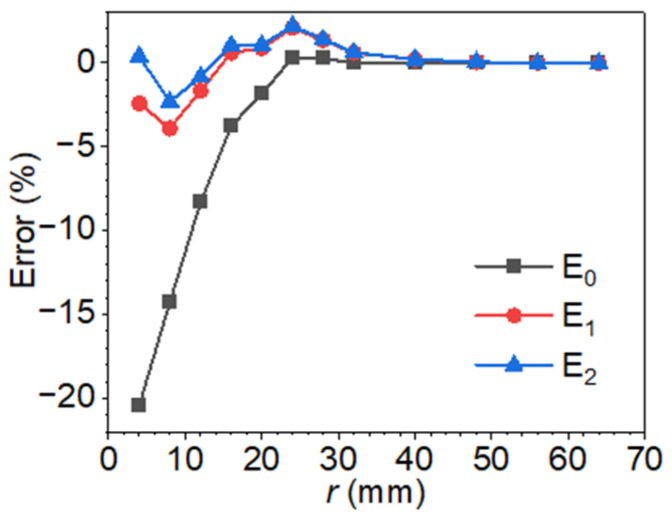
Depth estimation errors after applying the correction model.

As illustrated in Figure 13, the application of the proposed empirical bias correction significantly enhances the accuracy of defect depth estimation using the blind frequency method. The initial uncorrected estimates, denoted as *E*_0_, exhibit a substantial negative bias, particularly for defects with a radius of 4 mm and depth of 8 mm, as previously shown in Figure 10. Following the first correction iteration, the prediction errors denoted by *E*_1_ are markedly reduced across the full range of radii, with deviations constrained within ±5%. A second correction step provides only marginal improvement, typically within 1–2% of the first corrected value, demonstrating the rapid convergence and robustness of the proposed correction model. These results indicate that a single correction step is generally sufficient to achieve reliable and accurate depth predictions, thereby minimizing computational cost without sacrificing precision. The results collectively validate the correction model’s effectiveness, establishing it as a computationally efficient and accurate solution for blind frequency–based defect depth inversion.

It should be noted that the parameters in Equation (5) were derived from curve fitting of numerical simulation results obtained using titanium alloy specimens with cylindrical void defects. These coefficients accurately describe the depth estimation bias within the parameter range examined in this study; however, they are not universal constants. The specific values of the fitting parameters may vary with the material’s thermal properties, defect geometry, and boundary conditions. Therefore, recalibration of these parameters is recommended to maintain accuracy when applying the proposed correction model to different materials or defect types. Furthermore, the proposed correction model also presents potential economic advantages. Since it is implemented through data post-processing, it can be integrated into existing LIT systems without additional hardware, resulting in minimal implementation cost. By improving the accuracy of defect depth estimation, the approach may reduce inspection time and the need for repeated measurements, thereby enhancing overall efficiency. Moreover, the additional computational cost is expected to be negligible compared with experimental time. These features suggest that the proposed method is both cost-effective and scalable for practical nondestructive testing applications.

### 4.2. Analysis of the Effect of Radius on the Phase Difference Method

**Figure 14:**
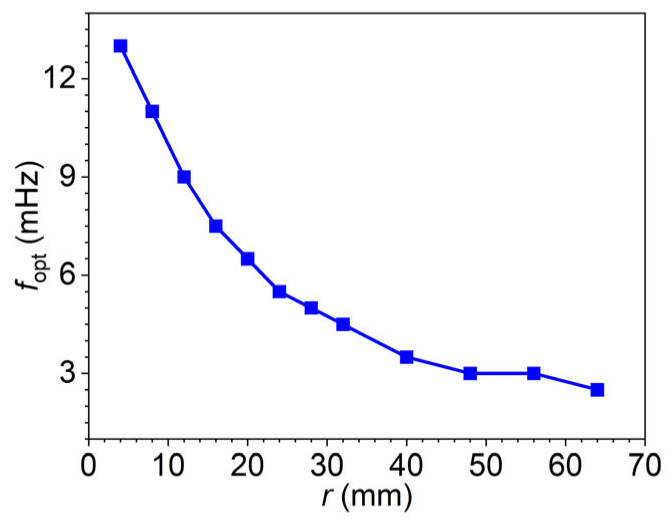
Optimal frequencies for models of different radii at a fixed depth of 8 mm [54].

The phase difference method estimates defect depth by analyzing the phase contrast between the surface temperature responses of defective and sound regions. Based on the finite element models introduced in Section 3.2, this section investigates how defect geometry and excitation frequency influence the accuracy of this method. To start with, models with a fixed defect depth of 8 mm and varying defect radii were simulated and analyzed. Figure 14 presents the corresponding optimal excitation frequencies extracted from the phase difference–frequency curves illustrated in Figure 2. These optimal frequencies serve as essential indicators for selecting appropriate inspection conditions. A well-chosen excitation frequency is critical to ensure sufficient phase contrast, which directly impacts the reliability of depth resolution and helps minimize estimation uncertainty.

**Figure 15:**
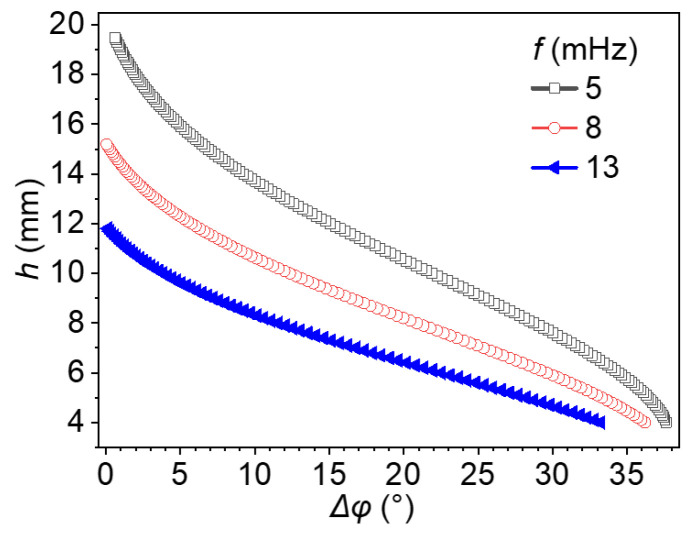
Defect depth–phase difference curves at varying excitation frequencies.

Figure 15 illustrates the relationship between *h* and phase difference (*Δφ*) at three excitation frequencies: 5, 8, and 13 mHz. These three representative cases were selected from Figure 4 because they provide distinct and monotonic phase difference–depth relationships within the relevant inspection range (6–10 mm), ensuring sufficient phase contrast for quantitative depth analysis. To ensure the reliability and physical validity of the analysis, only data points with positive phase differences were retained. The resulting *h − Δφ* curves demonstrate a clear frequency-dependent trend: for a given phase difference, higher excitation frequencies systematically yield smaller estimated defect depths. This inverse relationship is especially evident when comparing these three curves. Each frequency produces a distinct profile, and the shift becomes more significant as *Δφ* decreases. This behavior underscores the critical importance of using a reference *h-Δφ* curve that matches the excitation frequency employed in the inspection.

**Figure 16:**
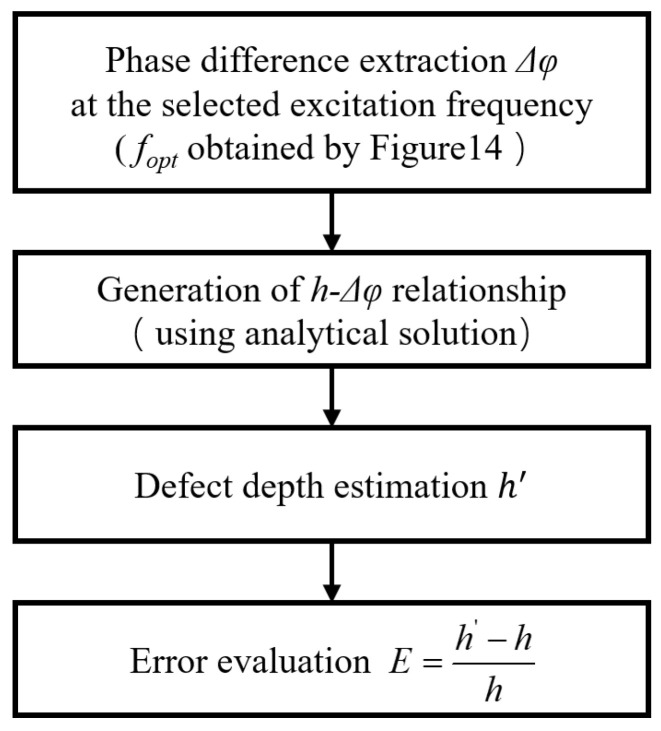
Flowchart of the phase difference–based depth evaluation.

Figure 16 presents the flowchart of the phase difference–based depth evaluation method. The procedure begins with the extraction of *Δφ* at the selected excitation frequency or optimal excitation frequency determined according to the radius of the defect, as illustrated in Figure 14. Next, the analytical solution is applied to generate the *h − Δφ* relationship, serving as a reference curve. Using this relationship, the defect depth (*h*′) is estimated from the measured phase difference. Finally, the estimation accuracy is quantified by evaluating the relative depth error, as shown in the flowchart. This workflow ensures a systematic approach for translating phase information into defect depth estimations.

**Figure 17:**
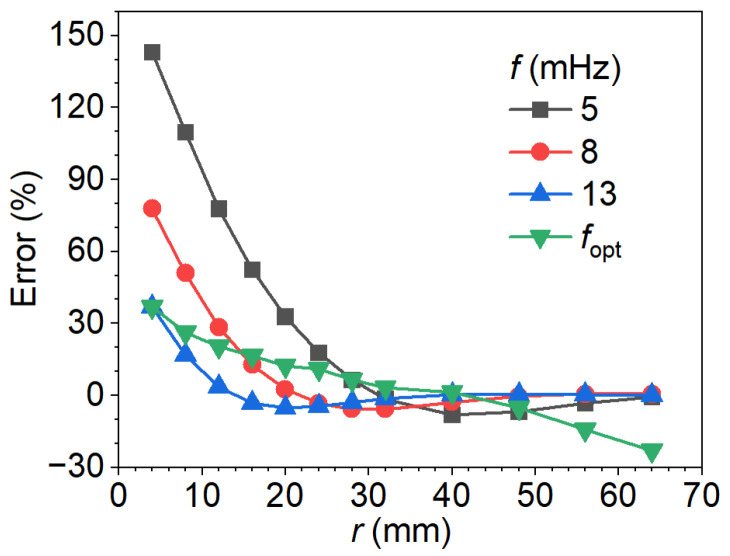
Depth estimation errors versus defect radius at fixed excitation frequencies and the optimal frequencies.

Figure 17 presents the defect depth estimates obtained at fixed excitation frequencies of 5, 8, and 13 mHz, as well as at the corresponding optimal excitation frequencies for each defect radius by the procedure shown in Figure 16. The estimation error shows a clear decreasing trend with increasing defect radius, gradually approaching zero for larger defects. This behavior suggests that the influence of lateral heat conduction diminishes as the defect radius increases. For defects with a small radius, the error is most significant, reaching 143% at 5 mHz, 78% at 8 mHz, and 37% at 13 mHz. And the error decreases systematically across all three frequencies with increasing radius. It is also noteworthy that the defect radius at which the error vanishes depends on the excitation frequency: the largest critical radius is observed at 5 mHz, whereas the smallest corresponds to 13 mHz. This frequency-dependent behavior can be attributed to variations in the thermal diffusion length. At lower frequencies, the longer diffusion length enhances lateral heat conduction, leading to larger errors. Conversely, higher frequencies reduce the diffusion length, thereby suppressing lateral conduction effects and improving depth estimation accuracy.

The optimal excitation frequencies corresponding to various radii (as identified in Figure 14), along with the calibration curve, were used in the phase difference method. The resulting depth estimations are presented by the green curve in Figure 17. For small defects, such as when the radius is close to 4 mm, the optimal frequency is 13 mHz, and the result coincides with that obtained from the 13 mHz curve. As the defect radius increases, the optimal frequency gradually decreases below 13 mHz, and the associated estimation errors remain slightly higher than those derived from the fixed 13 mHz calibration. Notably, when the radius exceeds 32 mm, the optimal detection frequency falls below 5 mHz. At such low frequencies, the thermal diffusion length becomes much larger, which enhances lateral heat conduction and leads to significant deviations in the estimated depth. For instance, at a radius of 64 mm, the optimal frequency drops to 2.5 mHz, resulting in an estimation error of approximately −23%, while the errors at 5, 8, and 13 mHz have already converged to near zero. The results emphasize the pronounced sensitivity of the phase difference method to both the frequency-dependent thermal diffusion length and the geometry of defects. In particular, larger-radius defects necessitate excitation at lower frequencies, which results in longer thermal diffusion lengths, thereby increasing the depth estimation errors.

**Figure 18:**
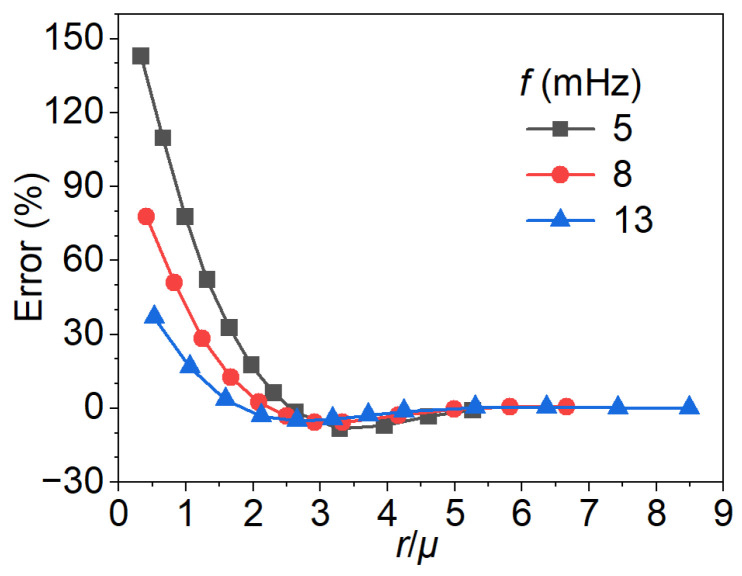
Depth estimation error as a function of the defect radius-to-thermal diffusion length ratio across varying excitation frequencies.

Figure 18 presents the depth estimation error as a function of the defect radius-to-thermal diffusion length ratio (r/μ) for excitation frequencies of 5, 8, and 13 mHz. The results indicate that the estimation error strongly depends on both the excitation frequency and the relative size of the defect with respect to the thermal diffusion length. At small ratios (r/μ<2), the error is significant, particularly at lower excitation frequencies, where values exceeding 100% are observed at 5 mHz. As r/μ increases, the error rapidly decreases and converges toward zero at the ratio of 1.5 for 5 mHz, 2 for 8 mHz, and 2.5 for 13 mHz. This behavior suggests that higher excitation frequencies facilitate more accurate depth estimation for defects with a small radius, while lower frequencies require proportionally larger defect-to-diffusion length ratios to achieve comparable accuracy. Where r/μ>4, error curves of all cases collapse onto a near-zero baseline, indicating that the defect size dominates over thermal diffusion effects, and the frequency dependence becomes negligible. These observations highlight *r*/*μ* as a dimensionless parameter for evaluating detection performance, while also emphasizing the distinct role of excitation frequency in the low *r*/*μ* regime.

**Figure 19:**
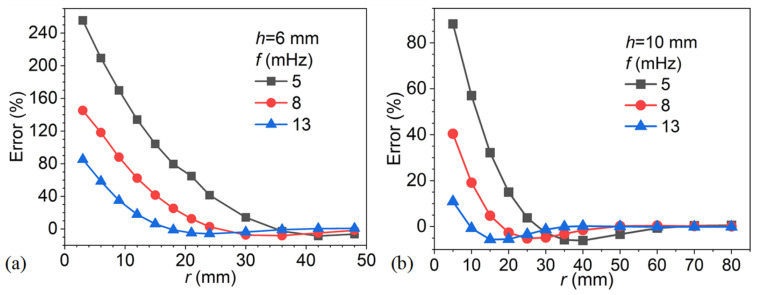
Depth estimation error as a function of defect radius for different excitation frequencies: (**a**) defect depth of 6 mm, (**b**) defect depth of 10 mm.

To further validate the generality of the observed trends, additional models with defect depths of 6 mm and 10 mm were analyzed, using the corresponding radii defined in Table 2. The results are presented in Figure 19, where subfigures (a) and (b) correspond to defect depths of 6 mm and 10 mm, respectively. In both cases, the error decreases monotonically with increasing defect radius, with apparent differences between excitation frequencies. At small radii, lower frequencies (e.g., 5 mHz) yield considerably larger errors, exceeding 200% in the 6 mm case and 80% in the 10 mm case, whereas higher frequencies (e.g., 13 mHz) show significantly reduced errors. As the defect radius increases, the error curves converge toward zero, with convergence occurring more rapidly at higher frequencies. These results confirm that the frequency-radius dependency observed in the 8 mm case (Figure 17) is consistently maintained across different defect depths, underscoring the robustness of the thermal diffusion influence on depth estimation by the phase difference method.

**Figure 20:**
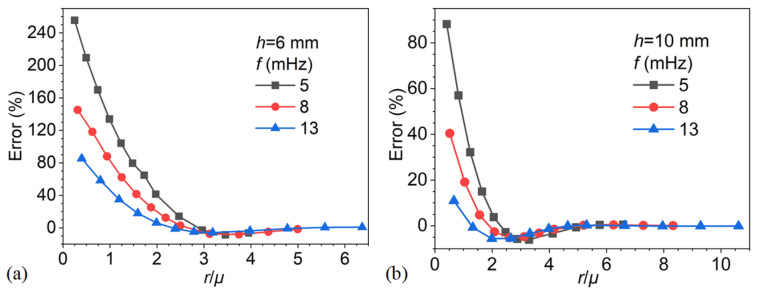
Depth estimation error as a function of *r*/*μ* for different excitation frequencies: (**a**) defect depth of 6 mm, (**b**) defect depth of 10 mm.

Figure 20 presents the depth estimation error as a function of the normalized parameter *r*/*μ* for defect depths of 6 mm and 10 mm at excitation frequencies of 5, 8, and 13 mHz. The plots clearly demonstrate that both excitation frequency and defect depth strongly influence the error behavior. At a small ratio (*r*/*μ* < 2), the errors are substantial, particularly at lower frequencies, where values exceed 200% for the 6 mm case and approach 80% for the 10 mm case. Higher excitation frequencies, such as 13 mHz, consistently yield reduced errors, confirming their greater suitability for resolving shallower or smaller defects. As *r*/*μ* increases, the errors decrease monotonically and converge toward zero. The critical value of *r*/*μ* at which convergence occurs, however, depends on the defect depth: shallower defects require proportionally larger values of *r*/*μ* to suppress the error. This trend indicates that normalized defect size, relative to thermal diffusion length, plays a critical role in error reduction, but the threshold for achieving acceptable accuracy shifts with defect geometry.

These observations reinforce the significance of *r*/*μ* as a generalized scaling parameter in the phase difference method. Nonetheless, the results also highlight that both the magnitude of error and the convergence threshold are governed jointly by excitation frequency and defect depth. In practical terms, this means that although lateral heat conduction effects become negligible once *r*/*μ* exceeds approximately 3–4, it is not feasible to define a universal error-correction rule based solely on the *r*/*μ* parameter. Instead, frequency and defect geometry must be considered together to ensure robust depth estimation. Overall, the findings reveal that while *r*/*μ* provides a useful framework for characterizing detection performance, the phase difference method remains highly sensitive to both excitation frequency and defect depth. These dependencies underscore the complementary insights gained when comparing phase difference analysis with blind frequency approaches.

### 4.3. Limitations and Future Work

Despite the valuable insights gained in this study, several limitations must be acknowledged to delineate the scope of applicability and guide subsequent research efforts. The present work primarily focuses on the theoretical and numerical investigation of quantitative depth estimation in LIT. While the analytical and finite element models were rigorously validated through convergence studies and parametric analyses, certain simplifications were necessary to isolate the effects of lateral heat conduction and excitation frequency. These include the assumptions of isotropic and homogeneous material properties, idealized thermal boundary conditions, and the exclusion of environmental noise conditions that may not fully reflect real-world engineering scenarios. In detail, the parameters of the proposed correction model were derived from simulations involving titanium alloy specimens with cylindrical defects. Although the model demonstrated strong performance under these conditions, its applicability to different materials, defect geometries, and thermal boundary conditions remains unverified. Moreover, this study did not consider the effects of surface emissivity variations and measurement noise, both of which can significantly influence signal intensity and introduce apparent phase shifts, especially under low signal-to-noise conditions, which may pose notable challenges in practical LIT applications.

In general, the correction model has not yet been experimentally validated under real inspection environments. Future research will address this by incorporating the effects of surface emissivity variability, noise, and irregular boundaries through both enhanced simulations and controlled experimental setups. In particular, LIT experiments will be conducted on titanium and composite specimens containing artificial subsurface defects to evaluate the model’s robustness and reliability under realistic conditions. Thus, the modeling framework will be extended to include anisotropic and multilayered materials to improve its relevance to composite structures commonly found in aerospace and energy systems. Sensitivity analyses will also be conducted to explore how the correction model parameters respond to variations in material and defect characteristics, thereby facilitating the development of a more generalized and adaptable correction framework for broader nondestructive testing applications. Finally, future efforts may focus on refining the phase difference method by introducing frequency-dependent correction models. This would complement the current blind frequency approach and offer improved accuracy in addressing the complex coupling among defect depth, excitation frequency, and defect geometry.

## 5. Conclusions

This study establishes a quantitative framework to improve subsurface defect depth estimation in lock-in thermography (LIT) by explicitly accounting for lateral heat conduction effects. Two inversion strategies, the blind frequency and phase difference methods, were analyzed using one-dimensional analytical and three-dimensional finite element models.

Results show that the blind frequency method underestimates defect depth by up to −20.7% when the radius-to-depth ratio (*r*/*h*) is 0.5, while the error becomes negligible once this ratio exceeds 3. An exponential correction model was developed to compensate for this bias, effectively reducing the estimation error to within ±5% after a single iteration. In contrast, the phase difference method shows stronger dependence on defect depth, excitation frequency, and the ratio of defect radius to thermal diffusion length, which complicates the development of a universal correction scheme.

Overall, this study identifies lateral heat conduction as a key source of bias and proposes a correction framework that is expected to significantly improve the quantitative accuracy of defect depth characterization based on the blind frequency method in LIT. The proposed approach may also provide a solid foundation for future extensions to anisotropic and multilayer materials.

## Figures and Tables

**Table 1:** Material parameters [54].

Material	Thermal Diffusivity (mm^2^/s)	Thermal Conductivity (W/(m·K))	Specific Heat Capacity (J/(kg·K))	Density (kg/m^3^)
Titanium	2.32	7	678	4450
Air	22.24	0.026	1007	1.161

**Table 2:** Defect depths and radii in finite element models.

Defect Depth *h* (mm)	Radii *r* (mm)												
	*r*/*h*	0.5	1	1.5	2	2.5	3	3.5	4	5	6	7	8
6		3	6	9	12	15	18	21	24	30	36	42	48
8		4	8	12	16	20	24	28	32	40	48	56	64
10		5	10	15	20	25	30	35	40	50	60	70	80
12		6	12	18	24	30	36	42	48	60	72	84	96
14		7	14	21	28	35	42	49	56	70	84	98	112
16		8	16	24	32	40	48	56	64	80	96	112	128

**Table 3:** Blind frequency and depth estimation error for different defect sizes.

Radius-to -Depth Ratio	Depth 6 mm			Depth 8 mm			Depth 10 mm			Depth 12 mm			Depth 14 mm			Depth 16 mm		
	Radii (mm)	*f*_blind_ (mHz)	Error (%)	Radii (mm)	*f*_blind_ (mHz)	Error (%)	Radii (mm)	*f*_blind_ (mHz)	Error (%)	Radii (mm)	*f*_blind_ (mHz)	Error (%)	Radii (mm)	*f*_blind_ (mHz)	Error (%)	Radii (mm)	*f*_blind_ (mHz)	Error (%)
0.5	3	82.1	−20.47	4	46.1	−20.45	5	29.7	−20.71	6	20.5	−20.53	7	15.1	−21.27	8	11.7	−20.68
1.0	6	71.5	−14.83	8	39.7	−14.28	10	25.2	−13.93	12	17.5	−14.00	14	12.8	−14.51	16	9.9	−13.87
1.5	9	62.7	−9.05	12	34.7	−8.31	15	22.4	−8.70	18	15.3	−7.97	21	11.2	−8.50	24	8.7	−7.82
2.0	12	56.9	−4.53	16	31.5	−3.77	20	20.1	−3.62	24	13.9	−3.35	28	10.2	−4.08	32	7.9	−3.36
2.5	15	53.7	−1.73	20	30.3	−1.88	25	19.0	−0.87	30	13.1	−0.40	35	9.6	−1.13	40	7.5	−0.39
3.0	18	52.5	−0.61	24	29.0	−0.30	30	18.5	0.46	36	12.8	0.80	42	9.4	0.08	48	7.3	0.83
3.5	21	52.4	−0.47	28	29.0	0.28	35	18.5	0.59	42	12.7	0.88	49	9.4	0.09	56	7.3	0.81
4.0	24	52.7	−0.80	32	29.2	−0.05	40	18.9	−0.61	48	12.8	0.57	56	9.4	−0.14	64	7.4	0.61
5.0	30	52.9	−0.99	40	29.2	−0.05	50	18.9	−0.61	60	12.9	0.29	70	9.5	−0.40	80	7.4	0.35
6.0	36	52.9	−0.99	48	29.2	−0.05	60	18.9	−0.61	72	12.9	0.29	84	9.5	−0.35	96	7.4	0.40
7.0	42	52.9	−0.99	56	29.2	−0.05	70	18.9	−0.61	84	12.9	0.29	98	9.5	−0.35	112	7.4	0.40
8.0	48	52.9	−0.99	64	29.2	−0.05	80	18.9	−0.61	96	12.9	0.29	112	9.5	−0.35	128	7.4	0.40

## Data Availability

The original contributions presented in this study are included in the article. Further inquiries can be directed to the corresponding author.

## Abbreviations

The following abbreviations are used in this manuscript:

NDT	Nondestructive testing
IRT	Infrared thermography
LIT	Lock-in thermography
1D	One-dimensional
3D	Three-dimensional
$P_{\mathrm{free}}$	Temperature measurement point in the defect-free region
Pd	Temperature measurement point above the defect center
